# Detection of *Chlamydia trachomatis* and *Neisseria gonorrhoeae* (and Its Resistance to Ciprofloxacin): Validation of a Molecular Biology Tool for Rapid Diagnosis and Treatment

**DOI:** 10.3390/antibiotics13111011

**Published:** 2024-10-28

**Authors:** María Paz Peris, Henar Alonso, Cristina Escolar, Alexander Tristancho-Baró, María Pilar Abad, Antonio Rezusta, Ana Milagro

**Affiliations:** 1 Institute for Health Research Aragon, 50009 Zaragoza, Spain; aitristancho@salud.aragon.es (A.T.-B.); arezusta@salud.aragon.es (A.R.); amilagro@salud.aragon.es (A.M.); 2 Department of Animal Pathology, Faculty of Veterinary Sciences, Universidad de Zaragoza, 50013 Zaragoza, Spain; 3 Department of Microbiology Paediatrics Radiology and Public Health, Faculty of Medicine, Universidad de Zaragoza, 50009 Zaragoza, Spain; henar83.alonso@gmail.com; 4 Department of Animal Production and Food Science, Faculty of Veterinary Sciences, Universidad de Zaragoza, 50013 Zaragoza, Spain; cristinaesco87@gmail.com; 5 Microbiology Unit, Miguel Servet University Hospital, 50009 Zaragoza, Spain; mpabada@salud.aragon.es

**Keywords:** sexually transmitted diseases, molecular diagnostic techniques, antimicrobial resistance, ciprofloxacin resistance

## Abstract

**Background and Objectives***: Neisseria gonorrhoeae* and *Chlamydia trachomatis* can cause similar clinical syndromes and may coexist in infections. In emergency medicine, empirical treatment targeting both pathogens is often employed, potentially contributing to antibiotic resistance. Gonococcal resistance has emerged against first-line antimicrobials, necessitating prior testing for fluoroquinolone susceptibility. Certest Biotec developed two molecular diagnostic products for simultaneous detection: VIASURE *C. trachomatis* & *N. gonorrhoeae* Real-Time PCR Detection Kit and VIASURE *Neisseria gonorrhoeae* Ciprofloxacin-Resistant Real-Time PCR Detection Kit. To evaluate these products, clinical performance assessments were conducted at the Clinical Microbiology Laboratory of Miguel Servet University Hospital in Zaragoza, Spain. **Results and Conclusions**: Both VIASURE assays under study demonstrated high clinical sensitivity and specificity compared to reference molecular assays and Sanger sequencing. These kits offer an accurate diagnosis, facilitating appropriate treatment choices while addressing concerns about emerging antibiotic resistance. **Methods**: A total of 540 clinical samples from 540 patients already characterized as positive or negative for CT and NG by a molecular assay and by antibiotic susceptibility testing for ciprofloxacin using a concentration gradient diffusion method were used for the clinical evaluation. In cases where sensitivity results were unavailable, conventional PCR and Sanger sequencing were employed.

## 1. Introduction

Sexually transmitted infections (STIs) are a public health problem worldwide in terms of both morbidity and mortality, mainly related to the complications and sequelae derived from inadequate diagnosis or treatment [1,2,3]. The most reported causal agents are *Chlamydia trachomatis* (CT) and *Neisseria gonorrhoeae* (NG), with 105.7 million and 106.1 million new cases each year, respectively [4].

*Chlamydia trachomatis* is a Gram-negative, intracellular obligate bacterium belonging to the Chlamydiaceae family and is the causal agent of several diseases in humans, including nongonococcal urethritis, cervicitis, salpingitis, pelvic inflammatory disease, trachoma, lymphogranuloma venereum, and neonatal eye infections [5,6]. Most genital chlamydial infections are often asymptomatic and can lead to complications if untreated [7]. Due to the microorganism’s inability to grow in culture, diagnostic methods are mainly focused on nucleic acid amplification tests (NAATs), and they are recommended for screening high-risk groups [8,9]. *Neisseria gonorrhoeae*, the causative agent of gonorrhea, is a Gram-negative diplococcus belonging to the Neisseriaceae family [10]. The infection can be asymptomatic, especially in women, posing challenges in early detection. Complications include pelvic inflammatory disease and infertility [1]. Culture and NAATs are diagnostic methods, with the latter preferred for its sensitivity. Screening is crucial for at-risk individuals, with sample types varying by gender and sexual practices [11,12].

Most Clinical Microbiology Laboratories employ various methods for diagnosing STIs. Historically, culture has been the reference standard for pathogen detection; however, it may yield false-negative results due to challenges in preserving the viability of organisms during transport and storage or due to prior antibiotic treatment [8,9,10]. While serological tests are useful for epidemiological studies, they often cannot differentiate between active and past infections in most cases [13,14]. Compared with other methods, molecular techniques provide faster results, have higher sensitivity and specificity, do not require viable organisms, and are, in fact, the only available method for non-culturable organisms [15,16,17,18].

Most guidelines advocate for a syndromic approach to STI diagnosis, including empirical treatment that covers both CT and NG upon clinical suspicion. These guidelines often include the use of a third-generation cephalosporin like ceftriaxone and a macrolide such as azithromycin [19,20,21,22]. This practice can lead to the appearance of antibiotic resistance that affects the treatment not only of STIs but also of other infectious diseases. Therefore, the simultaneous detection of these pathogens could allow an accurate diagnosis, as well as an adequate treatment choice [23,24,25].

Due to the rapid increase in the antimicrobial resistance of NG in the last few decades, compounds like sulphonamides, penicillin, narrow-spectrum cephalosporins, tetracyclines, macrolides, and fluoroquinolones are now considered inadequate therapies for this infection [19,21]. In recent years, the appearance of pan-resistant strains has complicated the treatment choice even more, with huge implications for public health [26,27]. Although the gold standard for determining antibiotic susceptibility is still culture-dependent (e.g., Epsilon test), techniques such as sequencing and the use of microarrays have been developed, allowing direct and culture-free analysis [10].

Antibiotic treatment has undergone constant changes due to the extraordinary ability of NG to develop resistance. Sulphonamides were introduced in 1936, and resistance to these drugs was first described in the 1940s; later, the emergence of resistance to penicillin and tetracyclines in the 1980s made their use inadvisable, and in 2007, the World Health Organization recommended against quinolone treatment for the same reason. Moreover, gonococcal resistance has even reached the current first-line antimicrobials, with reported strains resistant to cefixime and ceftriaxone [28]. Given the need to consider new anti-infective strategies, the use of ciprofloxacin has been reconsidered. The current recommendation is to administer this treatment only after prior testing for fluoroquinolone susceptibility [29]. Ciprofloxacin has several advantages, such as good tolerability with limited side effects, good urogenital and extragenital pharmacokinetics, and the genetic bases of its resistance being relatively easy to detect [23,30].

Several studies in Canada, Brazil, South Africa, and Switzerland have demonstrated that the Ser91 mutation in the gyrA gene is a sufficient marker for ciprofloxacin resistance in >99% of cases [31]. Consequently, the development of a molecular test capable of detecting this mutation and differentiating ciprofloxacin-susceptible strains from those resistant would provide a highly sensitive and rapid method, bypassing the need for bacterial culture [32,33]. 

In this context, CerTest Biotec developed two rapid molecular diagnostic products for CT and NG (and its resistance to ciprofloxacin) for commercialization in compliance with IVDR standards [34].

The objective of this retrospective, collaborative study was to assess the clinical accuracy of the VIASURE *C. trachomatis* & *N. gonorrhoeae* Real-Time PCR Detection Kit (CerTest Biotec S.L., Zaragoza, Spain) and the VIASURE *Neisseria gonorrhoeae* Ciprofloxacin-Resistant Real-Time PCR Detection Kit (CerTest Biotec, Zaragoza, Spain). The evaluation was conducted using the standard procedures of the Clinical Microbiology Laboratory (CML) at Miguel Servet University Hospital (Zaragoza, Spain), with Sanger sequencing serving as the reference method.

## 2. Results

### 2.1. Routine Clinical Microbiology Laboratory (CML) Diagnostic Methods’ Results

A total of 540 samples isolated as part of the routine diagnosis workflow of the CML between October 2019 and April 2023 were selected. Of those, 133 and 192 samples were positive for CT and NG detection, respectively. Coinfection with both microorganisms was detected in 14 specimens. The remaining 215 samples were negative. Routine clinical diagnosis results for these two microorganisms were based on PCR using the Allplex™ STI Essential Assay (Seegene^®^, Seoul, Korea) (Table 1 and Table 2).

Culture antibiotic susceptibility and sequencing were used as reference techniques for ciprofloxacin susceptibility. Initially, routine antibiotic (ciprofloxacin) susceptibility CML testing revealed 116 and 50 ciprofloxacin-resistant and susceptible NG samples, respectively. Regarding the 26 NG-positive samples without CML antibiotic susceptibility, 12 were ciprofloxacin-resistant and 12 ciprofloxacin-susceptible, as determined by sequencing. However, two samples could not be sequenced (Table 3) due to insufficient DNA yield. Both samples were excluded from the study due to the lack of reference characterization to be able to compare the VIASURE results. Therefore, a total of 128 samples were considered ciprofloxacin-resistant and 62 sensitive. 

### 2.2. VIASURE C. trachomatis & N. gonorrhoeae Real-Time PCR Detection Kit Evaluation

All real-time PCR curves from positive samples presented a sigmoidal shape with the three typical phases, i.e., baseline, logarithmic, and plateau phases. The assay yielded no abnormal amplifications. The internal control was amplified in all wells containing clinical samples. The positive and negative controls were amplified in all runs. One CT-DNA-positive sample for the Allplex™ STI Essential Assay was not detected when using the VIASURE Kit (CerTest Biotec, Zaragoza, Spain). The sample was reanalyzed using both techniques from the DNA extraction step, and the same results were obtained; thus, the result was considered a CT false-negative value of the VIASURE assay. Regarding NG results, no discrepant results were obtained (Table 4 and Table 5).

### 2.3. VIASURE Neisseria gonorrhoeae Ciprofloxacin Real-Time PCR Detection Kit Evaluation

The assay accurately detected all DNA samples that were positive for NG. No abnormal amplifications were observed, and all real-time PCR amplification curves from NG-positive samples exhibited the characteristic sigmoidal pattern consisting of the three expected phases: baseline, exponential (logarithmic), and plateau phases. However, VIASURE detected one NG-positive sample that the Allplex™ STI Essential Assay considered negative. No amplification was obtained using Sanger sequencing, and thus, the VIASURE result was considered a false positive for NG detection.

For ciprofloxacin sensitivity evaluation, two samples were excluded from the study due to the lack of DNA for obtaining the reference characterization to compare with the VIASURE results. A total of 190 samples were considered. All of them were previously reported as NG-positive specimens with Ct values in the Seegene PCR from 20.8 to 37.3. A subgroup of samples negative for NG and CT but positive for other ITS-related microorganisms was analyzed, with no non-specific amplification observed in the FAM or HEX channel. 

One discordant case of ciprofloxacin resistance was detected, being classified as resistant in the VIASURE PCR and susceptible in culture. Further characterization by Sanger sequencing revealed the presence of the marker variant, and thus, the result was treated as a true positive.

Conversely, in seven samples with only sequencing results, VIASURE did not detect either resistance to ciprofloxacin or the wild-type gene. Among these samples, six were confirmed as resistant through sequencing, while one was found to be sensitive (Table 6). 

## 3. Discussion

This retrospective study demonstrated the good clinical sensibility and specificity of two molecular assays with lyophilized and ready-to-use reagents for CT and NG detection and NG resistance to ciprofloxacin. An advantage of our study design was the use of real clinical samples from routine care in our CML, emulating the whole process from the beginning.

Sexually transmitted infections’ microbiological diagnosis is often complicated due to the fragility of the bacteria to withstand the environmental changes involved in sample acquisition and transfer to the microbiology laboratory, their demanding nutritional requirements for growth in culture, and the waiting times needed to obtain the antibiotic susceptibility profile [3,5,9,10]. All of these factors prevent rapid diagnosis and targeted treatment. The development of molecular tests has signified a paradigm shift, as they allow faster results to be obtained, have high sensitivity and specificity, and are applicable to polymicrobial samples or mixed infections. It is also possible to create circuits that allow the automation of processes and the analysis of a high volume of samples, which is an additional benefit from a healthcare point of view [21,36,37].

Ellis et al. (2019) found an interpretable susceptibility phenotype of 57.2%, taking into account the total number of samples analyzed [38]. Strikingly, in our study, the percentage of interpretable phenotypes was greater than 95%, which would allow medical decisions, especially regarding treatment, to be made without the need for additional tests in the vast majority of cases. Likewise, the concordance of PCR with standard culture or reference PCR results was quite high, similar to that obtained by Buckley et al. (2016) [39], who employed a similar study design. The VIASURE *Neisseria gonorrhoeae* Ciprofloxacin Real-Time PCR Detection Kit offers the added benefit of detecting NG in the ROX channel, reducing the likelihood of false positives and minimizing the number of tests performed and the turnaround time. This makes it a promising option for point-of-care use in high-volume hospital settings. Additionally, with a processing time of approximately two hours, this rapid qPCR tool could be effectively implemented to prevent unnecessary antibiotic treatments, thereby avoiding disruptions to the establishment of the microbiota early in life. Real-time PCR also boasts high specificity, a wide detection range, and the ability to detect single DNA copy differences, making it an ideal candidate as a rapid diagnostic test. Moreover, it aligns with the WHO’s ASSURED criteria, being affordable, sensitive, specific, user-friendly, rapid, robust, equipment-free, and accessible to end users, all while remaining environmentally safe and cost-effective [2,9,21,40].

Other studies have focused on developing solutions that include other resistance determinants in NG; however, they are usually more laborious and require more time in their elaboration and interpretation. In addition, resistance to third-generation cephalosporins is not currently a problem in our setting due to its low incidence. On the other hand, macrolide monotherapy is not recommended, so it is always necessary to test for fluoroquinolone susceptibility [31,41,42].

In this study, we showed that both VIASURE Kits present good clinical sensitivity and specificity compared to reference molecular assays results in a wide range of samples, including vaginal swabs, endocervical swabs, rectal swabs, urethral swabs, and urethral urine. 

It is worth noting that in seven samples where the VIASURE *Neisseria gonorrhoeae* Ciprofloxacin Real-Time PCR Detection Kit detected the presence of NG, there was no amplification detected in either the FAM (point mutation) or HEX (wild-type) channel. Out of these seven samples, six were found to be positive for mutations through sequencing. This could be attributed to the possibility that the kit does not detect all single-point mutations within the gene, potentially resulting in an underestimation of ciprofloxacin resistance. Since gonococcus did not grow in culture for these seven samples, an antibiogram could not be performed. According to the instructions for use, if no signal is detected in the FAM and HEX channels but a signal is present in the ROX channel, this may indicate a low concentration of DNA in the sample. In such cases, it is recommended to consider repeating the test. Employing a third molecular biology comparator could be a solution to address this discrepancy. 

Another point to bear in mind is the use of previously reported results (i.e., Clinical Microbiology Laboratory) for diagnostic purposes as comparators for assessing the presence of ciprofloxacin resistance. This carries with it the inherent biases of using retrospective data, and because not all diagnosed cases grew in culture, it is not possible to establish the susceptibility phenotype in all isolates. The use of Sanger sequencing helps overcome this issue, which is an advantage over other methods [43]; however, minor resistance mechanisms, such as mutations in *parC*, should be taken into consideration [33,44]. 

The evaluation of these products using curated collections of NG genomes, as described by Liu et al. (2022), could provide greater robustness and inter-comparability to the results, so it is an idea to be evaluated in the future [45]. Furthermore, considering the development of new products for diagnosing CT and NG, we would suggest a multiplex PCR that detects not only the bacteria but also their potential resistance to macrolides such as ciprofloxacin.

## 4. Materials and Methods

### 4.1. Study Design and Ethics Approval

This retrospective, comparative, and collaborative study aimed to evaluate the performance and clinical utility of the VIASURE *C. trachomatis* & *N. gonorrhoeae* Real-Time PCR Detection Kit and VIASURE *Neisseria gonorrhoeae* Ciprofloxacin-Resistant Real-Time PCR Detection Kit products using DNAs extracted from a total of 540 clinical samples from 540 different individuals. The reference diagnostic methods were those routinely used by the Laboratory of the HUMS: molecular diagnosis for the detection of CT and NG and a concentration gradient diffusion method on GC or chocolate agar medium using E-test strips for ciprofloxacin susceptibility detection. In addition, conventional PCR and Sanger sequencing were used as reference tests for 26 NG-positive samples, as the CML was unable to obtain an antibiotic susceptibility result. Sequencing was also used to resolve discrepancies between the CML and VIASURE kit results.

To perform this study, collaboration with the Biobank of the Aragon Health System was necessary. The Biobank carried out the correct management of clinical samples and personal data, ensuring the anonymization and correct traceability of the samples. The project followed the requirements of Spanish Policy for Biomedical Research 14/2007, of 3 July. The use of all data and samples was approved by the research ethics committee of Aragon (Comité de Ética de la Investigación de la Comunidad Autónoma de Aragón: CEICA) (Project license: PI22/400, date of approval: 5 October 2022).

### 4.2. Participants, Sample Size, Type of Clinical Samples, and Sample Collection

The evaluation was planned to be carried out with the remnants of clinical samples of patients (male and female adults) attended in centers and hospitals whose samples were referred to the CML from the Miguel Servet University Hospital, Zaragoza (Spain).

The inclusion criteria for sample selection were a sufficient sample volume to allow for evaluation and a positive or negative diagnosis made during routine laboratory procedures. The exclusion criteria considered whether the samples had been kept frozen until analysis and whether they had come from patients under the age of 18.

Out of the 540 specimens, 395 corresponded to men and 145 to women. The type of clinical samples included in the study were 48 urethral urine samples, 178 urethral swabs, 79 rectal swabs, 54 endocervical swabs/exudates, 106 oropharyngeal swabs, and 75 vaginal swabs. Samples were collected from October 2019 to April 2023 by trained personnel using sterile single-use Copan Liquid Amies Elution Swab (ESwab™ Copan, Brescia, Italy)) with flocked nylon filled with 1 mL of Liquid Amies preservation medium (COPAN innovation). The routine clinical diagnosis of these samples was carried out within the first 24 h after receiving the samples in the hospital’s microbiology department. Until this analysis, samples were properly stored at 4 °C. After routine clinical diagnosis, the remnants of the clinical samples were stored at −20 °C. The VIASURE analysis was conducted from April to August 2023. 

### 4.3. Routine Diagnostic Methods Used in the CML

The routine method for CT and NG diagnosis was based on the Seegene workflow. Initially, DNA extractions were performed using 200 μL of the clinical samples with the StarMag 96 × 4 Universal Cartridge (Seegene, Republic of Korea) into the Microlab STAR Let automatic extraction system (Hamilton, Switzerland) according to the manufacturer’s instructions. All nucleic acid elutions were performed in 100 μL of elution buffer. Then, the molecular assay Allplex™ STI Essential Assay (Seegene^®^) was performed. This assay can simultaneously detect 7 pathogens at the same time in a clinical sample: *Ureaplasma urealyticum*, *Neisseria gonorrhoeae*, *Mycoplasma hominis*, *Mycoplasma genitalium*, *Ureaplasma parvum*, *Chlamydia trachomatis*, and *Trichomonas vaginalis*.

The thermal profile protocol was as follows: step 1, 1 cycle of 50 °C 4 min; step 2, 1 cycle of 95 °C 15 min; step 3, 5 cycles of (95 °C for 30 s, 60 °C for 1 min, and 72 °C for 30 s); and step 4, 40 cycles of (95 °C for 10 s, 60 °C for 1 min, and 72 °C for 10 s). This analysis was performed using the CFX96 Real-Time PCR system (Bio-Rad^®^ Laboratories, Marnes-la-Coquette, France), and results were exported and analyzed in the specific software Seegene Viewer. For this comparative evaluation, data analysis was focused on CT- and NG-positive and negative values.

For antibiotic (ciprofloxacin) susceptibility, parallel evaluation was routinely performed using chocolate agar and/or NEISS agar culture media for NG detection. To observe colonies, the inoculated media were incubated for 24–48 h at 35 ± 2 °C in a CO_2_-enriched atmosphere. Identification was based on mass spectrophotometry (MALDI Biotyper^®^ sirius System, Bruker Daltonics, Billerica, MA, USA)). Then, a concentration gradient diffusion method on GC or chocolate agar medium using E-test strips was achieved. The cut-off points for susceptibility were those proposed by EUCAST clinical breakpoint tables v13.1 (≤0.03 mg/L sensitive, >0.06 mg/L resistant). These results were considered references for the calculation of the sensitivity and specificity of the test kit.

### 4.4. Assays Under Evaluation

VIASURE *C. trachomatis* & *N. gonorrhoeae* Real-Time PCR Detection Kit and VIASURE *Neisseria gonorrhoeae* Ciprofloxacin Real-Time PCR Detection Kit were performed with the sample leftovers after routine laboratory diagnosis. Following the Certest Biotec workflow, total DNA was obtained with the MagLEAD^®^ 12gC nucleic acid extractor (PSS instruments) using MagDEA^®^ Dx (Precision System Science^®^ Co., Ltd., Matsudo, Japan) reagents using 200 µL of the clinical sample, and the nucleic acids were eluted in a final volume of 100 µL of elution buffer.

#### 4.4.1. VIASURE *C. trachomatis* & *N. gonorrhoeae* Real-Time PCR Detection Kit

The VIASURE *N. gonorrhoeae & C. trachomatis* Real-Time PCR Detection Kit is a molecular assay designed for the simultaneous qualitative detection and identification of NG and/or CT DNA. It is suitable for use with clinical samples such as urethral urine, urethral swabs, rectal swabs, endocervical swabs/exudate, vaginal swabs, and oropharyngeal swabs from individuals suspected by healthcare professionals to have gonorrhea and/or chlamydia infections. According to the manufacturer’s instructions, pathogens were detected as follows: FAM for *C. trachomatis* and ROX for *N. gonorrhoeae*. The internal control was amplified in the HEX channel. The VIASURE kit batch used in this study was CTN112L-002, expiry date: 2024/04.

The thermal profile protocol was as follows: step 1, 1 cycle of 95 °C 2 min; steps 2 and 3, 45 cycles of 95 °C for 10 s, 63 °C for 50 s. This analysis was performed using the CFX96 Real-Time PCR system (Bio-Rad^®^ Laboratories, Marnes-la-Coquette, France), and results were interpreted by the CFX Manager ™ software (Version 2.1, Bio-Rad, Hercules, CA, USA).

#### 4.4.2. VIASURE *Neisseria gonorrhoeae* Ciprofloxacin Real-Time PCR Detection Kit 

The VIASURE *Neisseria gonorrhoeae* Ciprofloxacin Real-Time PCR Detection Kit is a molecular diagnostic tool designed for the qualitative detection and identification of NG DNA, as well as a specific point mutation in the gyrA gene. This mutation, which results in the substitution of serine at position 91 of the wild-type genotype with phenylalanine, confers resistance to ciprofloxacin in NG. The kit is intended for use with clinical specimens such as urethral urine and urethral, endocervical, vaginal, oropharyngeal, and rectal swabs from individuals suspected of having gonorrhea based on clinical evaluation by healthcare professionals. According to the manufacturer’s instructions, ciprofloxacin resistance was detected in the FAM channel, ciprofloxacin susceptibility in the HEX channel, NG presence in the ROX channel, and the internal control in the Cy5 channel. The VIASURE kit batch used in this study was NCR112L-001, expiry date: 2024/07. 

The thermal profile protocol was as follows: step 1, 1 cycle of 95 °C 2 min; steps 2 and 3, 45 cycles of 95 °C for 10 s, 63 °C for 50 s. Amplification was carried out in a CFX96 ™ real-time PCR system (Bio-Rad^®^ Laboratories, France). Data were interpreted by the CFX Manager ™ software. 

### 4.5. Bidirectional Sanger Sequencing

Bidirectional Sanger sequencing was used as a reference technique for the 26 NG-positive samples without CML antibiotic susceptibility results. It was also used to resolve discrepancies between the CML and VIASURE kit results.

Briefly, a 298pb region of the *gyrA* gene of NG was amplified using the primers previously described: CIP1-Fw 5′-GCGACGGCCTAAAGCCAGTG-3′ and CIP1-Rv 5′-GTCTGCCAGCATTTCATGTGAG-3′. The amplified region’s sequence has the mutation responsible for ciprofloxacin resistance in NG. Resistance to ciprofloxacin, in the case of NG, is due, in 99% of cases, to a point mutation located in the *gyrA* gene. The wild-type genotype has a serine residue at position 91 of the gene (S91), while the mutant genotype would have a phenylalanine in its place (S91F). In DNA bases, the change translates a “C” to a “T”.

### 4.6. Data Collection and Analysis

Data were collected in an Excel file, including all results. In addition, relevant clinical information was also included. Clinical sensitivity and specificity and negative and positive predictive values (with 95% confidence intervals), overall agreement, and likelihood ratios were calculated using the results of the Allplex™ STI Essential Assay (Seegene^®^), the CML’s routine antibiotic (ciprofloxacin) susceptibility testing, and sequencing as reference values, using the meta-DiSc^®^ version 1.4 freeware software [46]. Values were calculated after resolving discordant results to the maximum extent possible.

## Figures and Tables

**Table 1:** Reference diagnosis results for *Chlamydia trachomatis* yielded by Allplex™ STI Essential Assay (Seegene^®^).

Sample Type and Sex	*C. trachomatis*-Negative	*C. trachomatis*-Positive	Total
Endocervix	27	27	54
Woman	27	27	54
Oropharyngeal	103	3	106
Man	100	3	103
Woman	3	0	3
Rectal	52	27	79
Man	52	27	79
Urethra	153	25	178
Man	153	25	178
Urethral urine	34	14	48
Man	21	14	35
Woman	13	0	13
Vaginal	38	37	75
Woman	38	37	75
Total	407	133	540

**Table 2:** Reference diagnosis results for *Neisseria gonorrhoeae* yielded by Allplex™ STI Essential Assay (Seegene^®^).

Sample Type and Sex	*N. gonorrhoeae*-Negative	*N. gonorrhoeae*-Positive	Total
Endocervix	33	21	54
Woman	33	21	54
Oropharyngeal	92	14	106
Man	89	14	103
Woman	3	0	3
Rectal	60	19	79
Man	60	19	79
Urethra	46	132	178
Man	46	132	178
Urethral urine	44	4	48
Man	31	4	35
Woman	13	0	13
Vaginal	73	2	75
Woman	73	2	75
Total	348	192	540

**Table 3:** Reference diagnosis results for ciprofloxacin susceptibility in *Neisseria gonorrhoeae*-positive samples.

	Culture Antibiotic Susceptibility		Sequencing		
Sample Type	Resistant	Sensitive	Resistant	Sensitive	No Data
Endocervical	10	11	0	0	0
Oropharyngeal	0	0	8	6	0
Rectal	7	0	4	6	2
Urethra	96	36	0	0	0
Urethral urine	2	2	0	0	0
Vaginal	1	1	0	0	0

**Table 4:** Clinical sensitivity (SE), specificity (SP), positive and negative predictive values (PPVs and NPVs), overall agreement (OA), likelihood ratios (LRs), and strength of agreement (K) values for VIASURE *C. trachomatis* & *N. gonorrhoeae* Real-Time PCR Detection Kit compared to Allplex™ STI Essential Assay (Seegene^®^) for *C. trachomatis* evaluation.

Target	OA (%)	TP	TN	FP	FN	SE	SP	PPV	NPV	LR+	LR−	*K* *
Global	99.8 (98.9–100)	132	407	0	1	0.99 (0.95–0.99)	1 (0.98–1)	1 (0.96–1)	0.99 (0.98–0.99)	806 (50.55–12,878)	0.01 (0.002–0.05)	0.99
Endocervical samples	100 (93.4–100)	27	27	0	0	1 (0.84–1)	1 (0.84–1)	1 (0.84–1)	1 (0.84–1)	55 (3.52–858)	0.01 (0.001–0.28)	1
Rectal swabs	98.7 (93.2–99.8)	26	52	0	1	0.96 (0.79–0.99)	1 (0.91–1)	1 (0.84–1)	0.98 (0.88–0.99)	100.3 (6.34–1585)	0.05 (0.01–0.25)	0.99
Urethral swabs	100 (97.9–100)	25	153	0	0	1 (0.83–1)	1 (0.97–1)	1 (0.83–1)	1 (0.97–1)	302 (18.97–4810)	0.01 (0.001–0.3)	1
Urethral urine	100 (92.6–100)	14	34	0	0	1 (0.73–1)	1 (0.87–1)	1 (0.73–1)	1 (0.87–1)	67.6 (4.3–1062)	0.03 (0.002–0.51)	1
Oropharyngeal swab	100 (96.5–100)	3	103	0	0	1 (0.31–1)	1 (0.95–1)	1 (0.31–1)	1 (0.95–1)	182 (11.1–2962)	0.12 (0.009–1.67)	1
Vaginal swab	100 (95.1–100)	37	38	0	0	1 (0.88–1)	1 (0.88–1)	1 (0.88–1)	1 (0.88–1)	76.9 (4.9–1209)	0.013 (0.001–0.2)	1

* Strength of agreement was almost perfect according to Schober et al. (2018) [35] (*p* < 0.001).

**Table 5:** Clinical sensitivity (SE), specificity (SP), positive and negative predictive values (PPVs and NPVs), overall agreement (OA), likelihood ratios (LRs), and strength of agreement (K) values for VIASURE *C. trachomatis* & *N. gonorrhoeae* Real-Time PCR Detection Kit compared to Allplex™ STI Essential Assay (Seegene^®^) for *N. gonorrhoeae* evaluation.

Target	Overall Agreement	TP	TN	FP	FN	SE	SP	PPV	NPV	LR+	LR−	*K*
Global	100 (99.3–100)	192	348	0	0	1 (0.97–1)	1 (0.98–1)	1 (0.97–1)	1 (0.98–1)	696 (43.6–11,108)	0.003 (0–0.041)	1
Endocervical samples	100 (93.4–100)	21	33	0	0	1 (0.8–1)	1 (0.87–1)	1 (0.8–1)	1 (0.87–1)	66.4 (4.23–1041)	0.023 (0.001–0.35)	1
Rectal swabs	100 (95.4–100)	19	60	0	0	1 (0.79–1)	1 (0.92–1)	1 (0.79–1)	1 (0.92–1)	118 (7.51–1881)	0.02 (0.002–0.38)	1
Urethral swabs	100 (97.9–100)	132	46	0	0	1 (0.96–1)	1 (0.9–1)	1 (0.96–1)	1 (0.9–1)	93.6 (5.94–1475)	0.004 (0–0.06)	1
Urethral urine	100 (92.6–100)	4	44	0	0	1 (0.4–1)	1 (0.9–1)	1 (0.4–1)	1 (0.9–1)	81 (5.06–1295)	0.1 (0.007–1.4)	1
Oropharyngeal swab	100 (96.6–100)	14	92	0	0	1 (0.73–1)	1 (0.95–1)	1 (0.73–1)	1 (0.95–1)	179.8 (11.31–2857)	0.03 (0.002–0.51)	1
Vaginal swab	100 (95.1–100)	2	73	0	0	1 (0.19–1)	1 (0.93–1)	1 (0.19–1)	1 (0.93–1)	123.3 (7.43–2045)	0.16 (0.01–2.10)	1

**Table 6:** Clinical sensitivity (SE), specificity (SP), positive and negative predictive values (PPVs and NPVs), overall agreement (OA), likelihood ratios (LRs), and strength of agreement (K) values for VIASURE *Neisseria gonorrhoeae* Ciprofloxacin Real-Time PCR Detection Kit compared to reference technique (culture antibiotic susceptibility and sequencing).

Target	OA	TP	TN	FP	FN	SE	SP	PPV	NPV	LR+	LR−	*K*
*N. gonorrhoeae*	99.8 (99–100)	190	347	1 *	0	1 (0.98–1)	0.99 (0.98–1)	0.99 (0.96–0.99)	1 (0.98–1)	232 (46.9–1145)	0.003 (0–0.041)	0.99
Point mutations in the *gyrA* gene	96.3 (92.6–98.2)	129	55	0	6	0.95 (0.9–0.98)	1 (0.93–1)	0.99 (0.95–0.99)	0.9 (0.79–0.95)	106.7 (6.75–1684)	0.048 (0.02–0.1)	0.92
V wild-type region of the *gyrA* gene	96.3 (92.6–98.2)	61	128	0	1	0.98 (0.91–1)	1 (0.97–1)	0.9 (0.79–0.95)	0.99 (0.95–0.99)	251.8 (15.8–4006)	0.02 (0.005–0.11)	0.98

* No *N. gonorrhoeae* detection was obtained by sequencing.

## Data Availability

The data that support the findings of this study are available from M-P.P. upon reasonable request.
